# Sexuality and self-concept of morbidly obese women who are sexually attracted to men after bariatric surgery: a phenomenological study

**DOI:** 10.1186/s12905-024-03014-1

**Published:** 2024-03-13

**Authors:** José Granero-Molina, María del Mar Jiménez-Lasserrotte, Cristina Arias Hoyos, María José Torrente Sánchez, Cayetano Fernández-Sola, María Dolores Ruiz-Fernández

**Affiliations:** 1 Physiotheraphy and Medicine Department, University of Almería, Almería, Spain; 2 Faculty of Health Sciences, Universidad Autónoma de Chile, Chile, Santiago 7500000 Spain; 3 Bariatric Surgery, Andalusian Health Service, Almería, Spain; 4 Bariatric Surgery, Mediterraneo Hospital, Almería, Spain

**Keywords:** Bariatric surgery, Sexuality, Body, Obesity, Experiences

## Abstract

**Background:**

Morbid Obesity (MO) is a public health problem that affects a person’s physical, psychological and sexual well-being. Women with MO are affected by their body image and self-concept, and obesity stigma may affect women in social and sexual relationships.

**Objective:**

To describe and understand the experiences of morbidly obese heterosexual women (who are sexually attracted to men) in relation to their body image and sexuality after bariatric surgery.

**Methodology:**

Qualitative study using Merleau-Ponty’s hermeneutic phenomenology as a philosophical framework. Data collection took place between 2020 and 2021 in a southern Spanish province. A total of 22 in-depth interviews were conducted using open-ended questions until data saturation was reached.

**Results:**

Two main themes were identified: (1) “Escaping from a cruel environment”: weight loss to increase self-esteem; with the sub-themes: ‘I love myself now’, and ‘Body image and social relationships; a vicious circle; (2) “Now, I am truly me”: accepting my body to reclaim my sexuality, with the sub-themes: ‘The body as the focal point of sexuality’, and ‘When regaining your sex drive reignites your sex life and relationship’.

**Conclusion:**

Weight loss and body acceptance radically change morbidly obese women’s sex lives after bariatric surgery. They rediscover their bodies, have increased self-esteem, and see improvements in their social relationships and sexuality. These women feel seen, loved and desired, and now value their body image and femininity. As they go through continuous improvements following bariatric surgery, they gradually regain self-esteem, acceptance of their bodies and control over their sex life. Even though the women’s partners benefit from these improvements, they seem to be afraid of being left.

## Abstract (plain language summary)

Obesity is a problem that affects women’s physical, psychological and sexual well-being, as well as their social relationships. It is important to explore and understand the experiences of heterosexual women regarding their body and sexuality. After other treatments, women undergo surgery to reduce their obesity. After bariatric surgery women feel happier about themselves, experience less stigma and progressively recover their social and sex lives.

## Background

The World Health Organisation (WHO) defines being overweight as having a body mass index (BMI) ≥ 25 kg/m2, and obesity as a BMI ≥ 30 kg/m2 [1]. Morbid obesity (MO) is a serious health condition as it means having a BMI greater than 40 kg/m², combined with at least one serious obesity-related condition [2, 3]. MO is considered a public health challenge [4]; almost 40% of adults worldwide are overweight and almost 15% are obese [1, 5]. In Spain, 15.5% of women aged ≥ 18 years are obese [6]. MO is a precursor of high mortality [7], associated with comorbidities such as cardio-vascular disease, diabetes mellitus, hypertension, cancer, osteoarthritis, pelvic floor dysfunction or urinary incontinence [8, 9]. MO is also associated with psychological problems such as anxiety, depression, low self-esteem and disorders related to self-perception and body image [10]. This is compounded by social problems such as weight-related discrimination, sexual dysfunction and social stigma [11].

MO is associated with female sexual dysfunction (FSD), which is characterised by hypoactive sexual desire disorder, sexual aversion disorder, sexual arousal disorder, orgasmic disorder and sexual pain disorders [12]. This is in addition to disorders affecting libido, pleasure, reproduction [13, 14], intimacy, affection and self-care [15]. People with MO are affected in terms of self-perception and are dissatisfied with their body image, this is associated with worse mental and sexual health [7, 16]. Women with MO experience depression, anxiety, low self-esteem, limited acceptance of their body image and difficulty in interpersonal relationships. Negative correlations have been found between BMI, sex drive and the ability to experience orgasm [17]. Female sexuality is related to quality of life and body image, and a woman’s perception of her own body is associated with sexual satisfaction [18, 19]. Negative body image is linked to lower sexual activity, less satisfaction, poor communication and seeking professional help [20]. Women are a more vulnerable group because of society’s idealisation of their bodies.

The treatment of MO focuses on nutrition, physical exercise, lifestyle habits and surgical treatment. Bariatric surgery is an effective therapeutic measure for treatment of MO and its comorbidities, as it improves diabetes mellitus, dyslipidaemia and hypertension [11], as well as quality of life, sex hormones, fertility and sexual function [5, 11, 14, 15, 20]. Body perception and sex life are key factors in the decision to undergo bariatric surgery. Reviews suggest improved sexual function in women with MO after surgery [21], and almost half of patients dissatisfied with their sex life experienced improvements after surgery at their 5-year follow-up [22]. Body image also improved after bariatric surgery, although this is not always maintained over time [23]. Body image plays a relevant role in psychological disorders after bariatric surgery and is a key factor in the assessment and treatment of patients with MO [24]. Much of the motivation for people to opt for bariatric surgery is due to dissatisfaction with their appearance and body image [25]. The women’s motives for undergoing bariatric surgery stem from a desire to improve their fertility [26], as well as the expectation of better physical health, stronger personal identity, and improved social relationships; they consider surgery to be their last option [27]. Studies such as that of Mento et al. [28] recommend further research on self-concept and body image before and after bariatric surgery. In addition to epidemiological, nutritional, diagnostic or therapeutic studies, there is a need to understand morbidly obese heterosexual women’s experiences regarding their body image and sexuality after bariatric surgery [19, 29]. While the relationship between body image and sexual function has been examined in epidemiological or intervention studies, qualitative studies where women describe their own experiences are scarce [19]. Some studies have explored men’s and women’s experiences of MO prior to bariatric surgery [29, 30], but such experiences after surgery remain to be studied. Understanding these experiences could better prepare women for the life changes that follow bariatric surgery [31]. The aim of our study is to describe and understand the experiences of morbidly obese heterosexual women regarding their body image and sexuality after bariatric surgery.

## Methods

### Research design

This is a qualitative study that used Merleau-Ponty’s hermeneutic phenomenology as a framework. According to Merleau-Ponty, we perceive the world through our body [32], human consciousness is linked to corporeality, and the notion of ‘self’ is inextricably linked to our body and experiences. The participants live their experiences according to a body schema; we seek to understand the complexity of the phenomenon of sexuality, self-perception and body image in the different ways in which women with MO embody it. Merleau-Ponty advocates phenomenological reduction to capture experiences and the lifeworld (Lebenswelt) covering four existentials: lived space, lived body, lived time and lived relationships. In writing the manuscript, the Consolidated Criteria for Reporting Qualitative Research were applied (COREQ) [33].

### Participants and recruitment

The participants were selected through purposive sampling. A total of 22 women diagnosed with MO were recruited. The study took place in southern Spain between 2021 and 2022. Inclusion criteria: to be a heterosexual woman, aged 18 to 60 years, included in the Bariatric Surgery Programme, to have undergone surgery 2 or more years ago, to speak Spanish and to give informed consent. Exclusion criteria: a recent pregnancy, currently breastfeeding, going through menopause, previous bariatric surgery or refusal to participate in the study. The sociodemo-graphic characteristics of the participants are listed in Table 1.

**Table 1:** Sociodemographic data (N = 22)

Participant	Age	Onset of obesity	BMI prior to BS	BMI post-BS	Profession	Marital status
IDI1	33	Childhood	41.3	25.1	Farmer	Married
IDI2	35	Pregnancy	42.1	26.2	Shop assistant	Married
IDI3	48	Childhood	33.6	26.8	Unemployed	Single
IDI4	45	Adolescence	35.3	27.1	Chef	Single
IDI5	60	Adolescence	39.8	31.6	Teacher	Married
IDI6	53	Wedding	40.2	29.1	Consultant	Married
IDI7	54	Childhood	43.9	29.3	Unemployed	Single
IDI8	57	Childhood	44.3	29.8	Unemployed	Married
IDI9	32	Childhood	39,9	30.5	Beautician	Married
IDI10	45	Wedding	37.6	25.2	Healthcare	Married
IDI11	52	Pregnancy	51.2	42.3	Teacher	Married
IDI12	30	Childhood	39.8	26.1	Waitress	Partner
IDI13	36	Adolescence	38.4	24.2	Unemployed	Single
IDI14	48	Adolescence	37.1	29.5	Teacher	Married
IDI15	42	Pregnancy	36	24.3	Chef	Separated
IDI16	46	Pregnancy	35.9	22.8	Farmer	Married
IDI17	20	Childhood	40.5	35.6	Student	Single
IDI18	42	Adolescence	35.4	22.1	Civil servant	Single
IDI19	43	Childhood	35.1	26.5	Hairdresser	Married
IDI20	41	Adolescence	35.7	22.3	Architect	Married
IDI21	49	Wedding	50	34.7	Cleaner	Married
IDI22	30	Childhood	37.2	26.7	Bank teller	Single

IDI = In-depth Interview. BMI = Body Mass Index. BS = Bariatric Surgery

### Data collection

After obtaining permission, the researchers contacted the participants. The researchers knew the patients because they monitor them for up to 5 years post-intervention. Of thirty-four women with MO contacted, four did not answer the phone and eight refused to participate. In-depth interviews were conducted in which open-ended questions allowed for the women’s narratives to be told. Interviews, with an average duration of 52 min, took place in a room at the Department of Nursing, Physiotherapy and Medicine at the University of …., between November 2021 and April 2022. The interviews were conducted in Spanish by two researchers (M.J.T.S., C.A.H.) with more than 8 years of experience in bariatric surgery, which allowed for in-depth data collection. Before starting, the participants were given an explanation of the aim and ethical issues of the study, sociodemographic data were collected and informed consent was obtained. The interview began with an open-ended question: ‘What does the topic of sexuality after bariatric surgery suggest to you?’ The participants shared their experiences, and the interviewers took notes of verbal and non-verbal elements of communication. The interview ended with the question: ‘Do you have anything else to add?’ The researchers’ interpretations and reflections were continuously edited during the data analysis process. Data collection was stopped when data saturation was reached. Focus groups were ruled out as the participants refused to discuss their sexuality with other women.

### Data analysis

All annotations and in-depth interviews were transcribed and incorporated into a hermeneutic unit of the ATLAS programme. Ti 9.0. The analysis process was organised in three steps according to Merleau-Ponty’s phenomenology [34]: (1) Description: interviews were transcribed verbatim, transcripts were read and then re-read to understand the experiences of the participants. (2) Phenomenological reduction: a process of coding the transcripts without incorporating standpoints, concepts, memories or personal experiences of the researchers. (3) Phenomenological interpretation: the researchers develop themes and sub-themes drawn from the inductive data analysis to understand the study phenomenon. Analysis was conducted by the researchers who did not know the participants. Discrepancies in the analysis were discussed with the other mem-bers of the research team until a consensus was reached.

### Rigor

To ensure rigour, quality criteria were followed [35]. (1) Credibility: all phases of data collection are detailed, data interpretation followed a descriptive process verified by independent researchers (surgeon and nurse, bariatric surgery). (2) Transferability: exhaustive description of setting, participants, context and method. (3) Dependability: an expert not involved in data collection and analysis examined the results. (4) Confirmability: all researchers read the transcripts and agreed on the units of meaning, themes and subthemes.

### Ethical considerations

Permission was obtained from the Ethics and Research Committee of the Department of Nursing, Physiotherapy and Medicine of the University of Almeria (EFM/45/2018). The study complies with the requirements and ethical principles of the Declaration of Helsinki. Participants provided informed consent upon commencing the study.

## Results

A total of 22 women with MO who underwent bariatric surgery 2 or > years ago were interviewed. The mean age was 42.7 years, SD = 9.7. MO started during childhood for 40.9% of the participants, during adolescence for 27.3%, after getting married for 13.65%, and after pregnancy for 18.2%. The mean BMI was 39.1625 kg/m2 before surgery and 28.08 25 kg/m2 two or more years after bariatric surgery. 59.2% of the women were married, 31.8% were single, 4.5% were unmarried and 4.5% were living with a partner. Two main themes were extracted from the inductive data analysis: (1) “Escaping a cruel environment”: weight loss to increase self-esteem; and (2) “Now, I am truly me”: accepting my body to reclaim my sexuality (see Table 2).

**Table 2:** Themes, subthemes y units of meaning

Themes	Subthemes	Units of meaning
1.“Escaping a cruel environment”: weight loss to increase self-esteem	1.1 I love myself now	Reasons for the operation, changes, life changes, physique, self-esteem, negative body image
	1.2 Body image and social relationships: a vicious circle	Insecurity, idealisation of society, flattery, cruel environment, social relationships, new activities
2. “Now, I am truly me”: accepting my body to reclaim my sexuality	2.1 The body as the focal point of sexuality	Physique, clothing, lingerie, lights on, reduced mobility, self-esteem, embarrassment, new positions, nudity, self-care
	2.2 When regaining your sex drive reignites your sex life and relationship.	Changes in sex, attraction to others, orgasm, pleasurable sexual relations, new activities, sex drive, initiative, confidence

## “Escaping a cruel environment”: weight loss to increase self-esteem

The lives of women with MO change dramatically before and after undergoing bariatric surgery. A key aspect is increased self-esteem, which is linked to improved self-perception and social relationships. Two subthemes emerged.

### I love myself now

The participants highlighted two fundamental factors in their decision to undergo bariatric surgery: a feeling of self-consciousness brought about by not being comfortable with their own body, and an awareness of the deterioration of their health and quality of life due to MO. This is compounded by the aggravation of associated conditions, such as thyroid disease and difficulties in getting pregnant.

*I had surgery because I had a lot of complexes, because I had thyroid problems that were getting worse, and because I wanted to be a mother and I couldn’t because of my MO.*” (IDI1).

One of the most significant changes in women after bariatric surgery is the improvement in their self-esteem. The participants recognised that, prior to surgery, their attitude towards life was very negative; it affected the way they dressed, communication, leisure activities, establishing relationships and dating. Surgery changes their body physically and mentally, which gives them more confidence in decision-making.

I have learned to say yes to what I used to say no to. What does the operation change for you? Confidence, physique, will to live and health, … they’re all very important.” (IDI4).

Prior to surgery the participants rejected their own bodies; they did not look at themselves in the mirror because they did not like what they saw. They did not care about their appearance and neglected their sense of femininity. They did not find themselves attractive and did nothing to make others find them attractive either. One of the participants described her situation as follows:

*I saw this shapeless thing, an amorphous, ugly, very fat body with cellulite. I was disgusted to look at myself.”* (IDI5).

Surgery has many positive consequences. As one participant stated, she started to pay more attention to her physical appearance after the operation because she felt good about herself and her body. Likewise, the participants have better social and romantic relationships, and experience improvements in their overall quality of life. Now that they see a future that MO was hiding, they want to take care of their body and their appearance because it is an essential element of their new life.

*I like to get dressed up a lot more than I used to, …you feel prettier and better.*” (IDI2).

Another factor that helps them is the positive feedback from the people around them. According to the participants, since undergoing surgery, they get positive comments from others, which reaffirms their self-esteem and desire to improve every day.

*It motivates you, it makes you want to get dolled up. They say, “My goodness, look at you today!” That makes you feel motivated, not because you’re sexually interested in the men but because they’re looking at you, they’re telling you you’re beautiful…. tomorrow I’ll be even more beautiful!”* (IDI6).

### Body image and social relationships: a vicious circle

Social relationships are a fundamental aspect of people’s lives; the participants defined theirs as negative prior to bariatric surgery. At this stage, it is very difficult for them to relate to others, they lose their self-confidence, and insecurity isolates them socially.

*I used to be very withdrawn, I seemed to dislike everyone, I didn’t talk much, and, of course, you lose touch with people … You lose your self-confidence and, when you regain it and feel good, you also feel good around other people.*” (IDI7).

The participants said that contacting and interacting with people or social groups made them feel rejected, so they made excuses not to meet up with them. They felt ashamed and knew that they were the centre of attention and did not want to be. The participants have experienced stigma related to their MO, such as unpleasant jokes and mockery due to their physique. They have felt the accusatory gaze of a society that blames them for having become morbidly obese from not taking care of their health. Weight-related stigma isolated them and kept them stuck at home.

*I always made excuses, …that I didn’t feel like going, that I was tired, that I had a headache…a thousand excuses not to go.”* (IDI3).

Bariatric surgery has had a very positive effect on their willingness to interact with others. Improved self-image leads to improved self-esteem, which has a positive impact on recovering their social relationships:

*Now I feel more confident, I’m not ashamed to speak. When you are fat you are ashamed to talk, you think the others think: “look at what this fat woman is saying.”* (IDI4).

The women expressed how after surgery they wanted to take up activities that they had given up, such as physical exercise, outdoor activities and other hobbies. They have recovered their social relationships through spending time with friends, family or at patient associations.

*Now I feel like talking, being with people I know, meeting new people … I feel like going out more, having a coffee. When I was fat this didn’t happen, I feel like a new person and yes, …. I want to say it, I want you to see it!”* (IDI8).

Society’s cruelty is a factor that has caused them to have negative attitudes towards others. Most of the participants have had negative experiences due to comments about their body image or being made to feel uncomfortable. Society seems to deny them the right to be attractive to others and to be able to choose a partner. The women still have painful memories of these experiences, but they no longer suffer.

*They told me: “as you’re fat, you can’t expect to find the partner you want… you have to settle for whatever comes along, even if it’s not someone you really like”. All that … it’s all nonsense, but it gets stuck in your head and it wears you down.”* (IDI14).

The participants say that after surgery and weight loss, everything changes. They now receive compliments and positive comments from most people. This helps them to open up and relate to others. This is how one participant put it:

*They start by looking at you, then they say: “how beautiful, I don’t know what you’ve done to yourself…”. And maybe one of them is saying something to you and another one comes along and says: “it’s true, she’s very pretty”. It puts a smile on your face.”* (IDI20).

The women reaffirmed that bariatric surgery has radically changed their lives. They have higher self-esteem and confidence because of a transformation in their relationships and sexuality. The women with partners feel that they can respond to their partners’ sexual needs, that their husbands look at them lustfully, and they are proud of that.

*Yes, my husband now suggests going out for dinner, dancing, sexy clothes … everything has changed. I always used to be hidden, in the background… I didn’t want him to see me naked.”* (1DI16).

But despite improvements after bariatric surgery, the stigma of obesity does not disappear completely, and is still present. The women continue to receive negative comments after having undergone successful surgery. They recognise that it no longer affects them as much, but they feel the social stigma. The notion of an ideal body shape continues to influence their social and intimate relationships. As one participant said, she gets negative comments from those around her even after having lost weight. It is as if society does not accept the change and expects her to become morbidly obese again in the near future.

*Some people say: “keep your clothes, you’ll need them” and those words stick in your head, they are cruel, they hurt you deeply …. Puff.”* (IDI12).

## “Now, I am truly me”: accepting my body to reclaim my sexuality

Improvements in body self-perception and increased self-esteem directly influence the sexuality of women who undergo bariatric surgery. Two subthemes were developed from the data analysis.

### The body as the focal point of sexuality

The participants reported that the operation was a turning point in their sexual relations. Prior to bariatric surgery, the women with MO had serious mobility impairments, and symptoms of asphyxia, dyspnoea, tiredness and excessive sweating. This caused discomfort for them and their partners during sexual intercourse. After bariatric surgery, the women saw improvements in their sex life and relationships. They broke the monotony of always having sex in the same position, in the same places and under the same circumstances. This is what one woman said:

*“Before, you couldn’t move, you just went along with it, you weren’t active …. Now you are active, you can move, you feel good and you work out what you like. You get on top, on the bottom, on the side…”* (IDI16).

*“Before, sex could only be in bed. Now we change positions, places … everything. What my husband likes the most is for me to get on top of him, … that was unthinkable before. Now I can, I can get on top, I can enjoy it*!” (IDI21).

Most of the participants acknowledged that before the surgery they avoided sexual intercourse because they were ashamed of exposing their bodies and being touched. They all agreed that having sex with the light on was impossible, but this was no longer the case after the operation. In addition, they also claimed that their partners have noticed this positive change.

*Before, I didn’t like to be touched, … I was ashamed. Now I like him to touch me, to caress me, for it to last for a long time, everything is different, and he tells me so.”* (IDI1).

*“You feel much better (ha, ha … you don’t mind if the light is on), whether it’s in the evening or in the morning … Before it had to be at night, with the light off. Why? Because of my body weight, because I looked horrible, … he also saw it even though he didn’t tell me so.”* (IDI2).

After bariatric surgery, the participants stated that they no longer felt ashamed to be naked in front of their partners. Many of them agreed that even the skin that is visible due to weight loss does not bother them during sexual intercourse.

*“Before, I didn’t undress in front of my husband, now I look forward to being naked, I want my partner to be excited by my body, ….”* (IDI15).

*“The skin flaps are visible, I don’t like it, it’s a memory of what I was, …but they’re obvious, it’s as if you don’t see them.*” (IDI12).

Before the intervention, the women did not care about other aspects such as clothing or lingerie, which they struggled to find in their size; this is no longer a concern for them. Their body image is crucial to them now as it helps them to increase their sex drive and have sexual relations.

*“I didn’t buy clothes before. I never went to the shops, my mother would bring me clothes and tell me what looked good on me, … and that was it. Now, since I had the operation, I have started to lose weight and the dressing room seems too small”* (IDI18).

*“I can wear lingerie now, I go to the shops now. I look around, I can choose things for myself. I want to buy things because I’m going to wear them, they fit me well. I’ll wear this tonight, then I’m going to take a shower and wait for him (partner) in the bedroom…. I’m doing a lot of things I’ve never done before.”* (IDI14).

### When regaining your sex drive reignites your sex life and relationship

The participants confessed that their sex drive was very low before the operation, blaming their lack of confidence and self-esteem. Even in an intimate setting with their partners, they did not feel comfortable revealing their bodies. Their partners accepted them and wanted sex, but the women had no sex drive; the thought of having sex was repulsive to them.

*That idea is in your head… Even if your partner is telling you: “it’s me and I love you like this (with MO), who are you going to trust more? But you don’t… you close yourself off and you lose all desire.”* (IDI19).

The women described a shift in their sex drive after bariatric surgery, which was linked to changes in their body image and self-concept. Low libido even led some participants not to have sex for years or even throughout their lives, until they underwent surgery and lost weight.

*““I have never had a partner. I have had some opportunities, but as I didn’t love myself and I wasn’t comfortable with myself, if a man approached me, I would pull back. I wasn’t ready to discover more, … I lost my virginity at the age of 40.”* (IDI7)”.

The women described how their sex drive was reawakened after bariatric surgery. The participants with partners have noticed that both of them have increased libido, which directly improves their sex lives.

*“You feel great and you see that he looks at you differently, he’s more sexually at- tracted to you. Although he used to tell me that he loved me and that I was attractive, … I didn’t quite believe him. Yes, he loves you, he loved you with MO, but now with a better body you are more beautiful, more attractive, you feel better… he also has more sexual appetite, more desire to touch you, … you notice it and it’s nice*.” (IDI9).

One aspect that has improved considerably is taking initiative in sexual relations. Before the surgery, the women recognised that they were incapable of taking the initiative, suggesting sex games or introducing changes in their sex life. After gaining confidence and increased libido, they are looking to make changes, to innovate, to know what they want and to suggest it to their partners.

*Now I initiate things, and it’s clear that he (partner) wants to (have sex). In the past, he didn’t feel like it because he was always taking the initiative. I said no a lot and he was getting tired of it, … that has changed completely. Now I say what I want and what I don’t like.”* (IDI11).

The participants expressed how they did not fully enjoy sexual intercourse before surgery and often did not experience pleasure. The women mentioned having painful sexual experiences, and frequently not reaching orgasm. One participant spoke of how she would fake orgasms to avoid her partner feeling uncomfortable.

*It’s complicated, he wants to satisfy you, but you can’t, you don’t feel like it and you don’t have orgasms. It often bothered him (partner), he felt inadeqate as a man. I won’t deny it, … I have faked it because I was afraid of losing my partner*.” (IDI6).

This situation changes radically after surgery; the women now achieve a state of relaxation and confidence that allows them to enjoy sex more. Some participants acknowledged that they have been able to deal with problems in their sex life with their partner that they were unable to solve before.

*You are always thinking about not lying this way, that way, … I was always tense. When I was fat, I rarely ever reached orgasm with my partner. More than enjoying it … you are thinking about when it’s going to end.”* (IDI5).

Improved body image boosts both partners’ sex drive. These improvements go beyond penetrative sex and have an impact on other sexual behaviours such as masturbation. In addition, improve-ments in female sexuality have a positive impact on the relationship when the man also has problems due to andropause or other sexual dysfunctions.

*He had a (sexual) problem before my operation… Although penetration is still difficult, we masturbate, do our own thing and have a good time. It’s not just about penetration, there’s more to it, I go for it and, as he sees me feeling better about myself, he’s encouraged to do it.”* (IDI3).

Another noteworthy aspect are the changes that are brought about by improved self-confidence. Some of the women become empowered after bariatric surgery; they feel more attractive, want to be more feminine and attract men. This situation can change the status of their relationships, occasionally leading to break-ups or their male partners feeling jealous.

*You suddenly feel pretty, you are noticed, you are liked, you go out and socialise more …. It is difficult for both you and your partner to deal with this situation. He doesn’t always handle it well, he is not used to people looking at me like that (with desire). Yes, it can lead to arguments and problems. If the couple does not have a solid relationship, you can lose it all*.” (IDI15).

## Discussion

The aim of our study was to describe and understand the experiences of morbidly obese heterosexual women regarding their body image and sexuality two or more years after bariatric surgery. Our results show that the changes in the participants’ sex lives are positive for both themselves and their partners. Merleau-Ponty’s phenomenology has allowed us to explore the self-perception of the body and its influence on these women’s sexuality and life, confirming experiences in lived space, body, time and relationships [32]. According to Nilsson-Condori et al. (2020), a key factor in women with MO opting for surgery is the associated improvement in self-concept and self-esteem [36]. Additional motives for our participants, previously described by Paul et al. (2023) and Cohn et al. (2019), were to better their physical health and social relationships [26, 27]. In line with other studies [17, 37], our results confirm that weight loss after bariatric surgery improves the perception of sexual function, body image satisfaction and self-esteem in women with MO [38, 39]. Our results contrast with the study by Abdelsamea et al. (2023), who associate weight loss with psychological and quality of life improvements, but not with improvements in female sexual dysfunction [40]. Consistent with our findings, weight reduction after surgery boosts confidence and self-esteem, thereby improving social interactions [29, 41]. In line with the study by Wingfield et al. (2016), two years after bariatric surgery, the patients rediscover themselves and seem to regain lost hope [42]. Although some women suffer postoperative anxiety [22], our results do not reflect this, perhaps because several years have passed since the intervention. Body image and physical appearance are fundamental in the process; surgery results in temporal, spatial and relational changes in sexuality [32]. According to our results, the process is not static; surgery is not the end of the journey with MO, but rather the beginning of a new journey that involves sexuality. Women with partners acknowledge having support before and after surgery, and their partners also benefit from their improved sexuality [18]. Our results regarding the women’s partners are ambivalent, because while they seem to experience improvements in their sex life after the women undergo bariatric surgery, female empowerment leads the men to feel insecure. Indeed, studies such as that of Braming et al. (2021) have found that bariatric surgery is associated with increased likelihood of finding a partner for single people and increased risk of separation from a partner for those in a relationship [43]. Our participants agreed that the improvement of female self-concept and body image can lead to a lack of trust in their relationship as the men fear that their partners will leave them [17]. Our findings on the participants’ experiences corroborate that bariatric surgery improves sexual activity, sex drive, orgasm, intimacy, mobility, variety of sex positions and games [11, 21, 36, 37, 42, 44]. Even negative effects, such as excess skin, seem to be overlooked by the women in our study [45]. Understanding the concept of poor body image in bariatric surgery patients allows healthcare providers to provide preoperative preparation and postoperative interventions [46]. Implementing psychological care focusing on body functionality [28, 39, 47], promoting sex education and encouraging self-care [48], could be the key to a long-term recovery of morbidly obese women’s well-being, body image and sexuality after bariatric surgery. We concur with Lindberg et al. (2022) that the partners’ preparation for the life changes that arise after bariatric surgery may not be adequate; more time and a dialogic approach are needed for each individual to be able to manage and improve their sexual health [31]. Our study is one of the few that addresses the sexuality of women with MO after bariatric surgery, focusing on corporeality and their own experiences. This is a strength as women suffer from the social stigma of being overweight and struggle to talk about their sexuality, making it difficult to get them to participate in qualitative studies.

### Limitations

We acknowledge several limitations. Part of our sample was collected from a private hospital so women with fewer financial resources could be under-represented. The women knew one of the researchers; although it led them to agree to participate in the study, their accounts could have been biased. Part of our sample also participated in previous studies on experiences in the pre-operative phase. The more time that passes after bariatric surgery, the more the participants’ experiences of their sexuality can change. Therefore, findings may differ at much later stages. Our results only reflect the experiences of women who are sexually attracted to men; conclusions cannot be extended to other female sexual identities or partners.

## Conclusion

Sexual health has been shown to be key in improving the quality of life of women with MO after bariatric surgery. Weight loss and improved body image change the lives of these women; reconciliation with their bodies is linked to improvements in perceived self-esteem, communication and social relationships. The participants stated that since bariatric surgery they are once again able to enjoy a sense of sexuality they thought they had lost. The progressive increase in their self-confidence and desire is empowering. The women regain initiative and control of their sex lives, which has a direct impact on their social and sex lives, as well as on those of their partners. Going from a life as an obese person to having a radically slimmer body brings them face to face with an unexpected reality of mixed consequences. Despite the improvements they experience following bariatric surgery, they still have to deal with the stigma of being overweight and the impact it has on their relationships. This study is an exploration of the experiences of women with MO who have undergone bariatric surgery in relation to their sexuality. Further research could delve into these experiences at later stages among women with different sexual identities. There is also potential to develop interventions for improvement or to study the partners’ perceptions of changes in their social and sex lives.

### Implications for practice

Changes in body image, sexuality and social relationships should be addressed systematically by healthcare providers throughout the care of women with MO after bariatric surgery. Sexuality should be incorporated into care protocols and clinical practice guidelines right from when women are admitted into bariatric surgery programmes, given that progressive changes in post-surgical stages call for more long-term follow-up and support. The evolution of female sexuality after bariatric surgery affects both the individual and her partner; understanding the experiences of these women is key for designing specific interventions to provide coping strategies and support, which could benefit from including their partners.

## Data Availability

The datasets generated and/or analysed during the current study are not publicly available due to confdentiality but are available from the corresponding author on reasonable request.

## Declarations

The study protocol was approved by the University of Almería Ethics and Research Committee.

## References

[1] WHO (World Health Organization). Obesity and Overweight. WHO, Geneva, 2020.

[2] Mechanick JI, Kushner RF, Sugerman HJ, Michael, Collazo-Clavell ML, Spitz AF (2009). Medical guidelines for clinical practice for the perioperative nutritional, metabolic, and nonsurgical support of the bariatric surgery patient. Obesity.

[3] Mechanick JI, Youdim A, Jones DB, Garvey WT, Hurley DL, McMahon MM (2013). Clinical practice guidelines for the perioperative nutritional, metabolic, and nonsurgical support of the bariatric surgery patient – 2013. Surg Obes Relat Dis.

[4] Liu S, Cao D, Ren Z, Li J, Peng L, Zhang Q (2020). The relationships between bariatric surgery and sexual function: current evidence based medicine. BMC Urol.

[5] Mazer L, Morton JM, Reavis KM, Barrett AM, Kroh MD (2018). The obesity epidemic. Editors. The SAGES manual of bariatric surgery.

[6] INE (Instituto nacional de estadística. National Institute of statistics). Encuesta europea de salud en España 2020 [European Health Survey in Spain 2020]. 2020. https://www.ine.es/ss/Satellite?L=es_ES&c=INESeccion_C&cid=1259926457058&p=1254735110672&pagename=ProductosYServicios%2FPYSLayout. Accessed 4 Octr 2023.

[7] WHO (World Health Organization). 2023. WHO acceleration plan to stop obesity. https://iris.who.int/bitstream/handle/10665/367784/9789240073234-eng.pdf?sequence=1. Accessed 2 oct 2023.

[8] Sfahani SB, Pal S (2019). Does metabolic syndrome impair sexual functioning in adults with overweight and obesity?. Int J Sex Health.

[9] Rowland DL, McNabney SM, Mann AR (2016). Sexual function, obesity, and weight loss in men and women. Sex Med Rev.

[10] Taskin F, Karakoc A, Demirel G (2019). The effect of body image on sexual quality of life in obese married women. Health Care Women Int.

[11] Loh HH, Shahar MA, Loh HS, Yee A (2022). Female sexual dysfunction after bariatric surgery in women with obesity: a systematic review and meta-analysis. Scand J Surg.

[12] Deniz A, Okuyucu M (2022). The impact of obesity on fertility and sexual function in women of child bearing age. J Obstet Gynaecol.

[13] Jans G, Matthys C, Bel S, Ameye L, Lannoo M, Van der Schueren B (2016). AURORA: bariatric surgery registration in women of reproductive age - a multicenter prospective cohort study. BMC Pregnancy Childbirth.

[14] Salari N, Hasheminezhad R, Sedighi T, Zarei H, Shohaimi S, Mohammadi M (2023). The global prevalence of sexual dysfunction in obese and overweight women: a systematic review and meta-analysis. BMC Womens Health.

[15] Ferrández A, Novella B, Khan KS, Zamora J, Jurado AR, Fragoso M, Suárez C (2023). Obesity and female sexual dysfunctions: a systematic review of prevalence with meta-analysis. Semergen.

[16] Ivezaj V, Grilo CM (2018). The complexity of body image following bariatric surgery: a systematic review of the literature. Obes Rev.

[17] Pichlerova D, Bob P, Zmolikova J, Herlesova J, Ptacek R, Laker MK, Raboch J, Fait T, Weiss P (2019). Sexual dysfunctions in obese women before and after bariatric surgery. Med Sci Monit.

[18] Frederick DA, Gordon AR, Cook-Cottone CP, Brady JP, Reynolds TA, Alley J, Murray S (2022). Demographic and sociocultural predictors of sexuality-related body image and sexual frequency: the U.S. Body Project I. Body Image.

[19] Thomas HN, Hamm M, Borrero S, Hess R, Thurston RC (2019). Body image, attractiveness, and sexual satisfaction among midlife women: a qualitative study. J Womens Health.

[20] Ramseyer V, Ruhr L, Pevehouse D, Pilgrim S (2018). Exploring body image, contraceptive use, and sexual health out-comes among an ethnically diverse sample of women. Arch Sex Behav.

[21] Gao Z, Liang Y, Deng W, Qiu P, Li M, Zhou Z (2019). Impact of bariatric surgery on female sexual function in obese patients: a meta-analysis. Obes Surg.

[22] Steffen KJ, King WC, White GE (2019). Changes in sexual functioning in women and men in the 5 years after bariatric surgery. JAMA Surg.

[23] Bosc L, Mathias F, Monsaingeon M, Gronnier C, Pupier E, Gatta-Cherifi B. Long-term changes in body image after bariatric surgery: An observational cohort study. PLoS One. 2022; 7;17(12):e0276167. 10.1371/journal.pone.0276167 PMC9728839 36477002

[24] Campedelli V, Ciacchella C, Veneziani G, Meniconzi I, Paone E, Silecchia G, Lai C (2022). Body image and body mass index influence on psychophysical well-being in bariatric patients: a cross-sectional study. J Pers Med.

[25] Makarawung DJS, Monpellier VM, van den Brink F, Woertman L, Zijlstra H, Mink AB, van Ramshorst B, Geenen R (2020). Body image as a potential motivator for bariatric surgery: a case-control study. Obes Surg.

[26] Paul R, Drott J, Olbers T, Frisk J, Andersson E (2023). Motherhood and motivations for bariatric surgery - a qualitative study. Hum Fertil (Camb).

[27] Cohn I, Raman J, Sui Z (2019). Patient motivations and expectations prior to bariatric surgery: a qualitative systematic review. Obes Rev.

[28] Mento C, Silvestri MC, Muscatello MRA, Rizzo A, Celebre L, Cedro C, Zoccali RA, Navarra G, Bruno A (2022). The role of body image in obese identity changes post bariatric surgery. Eat Weight Disord.

[29] Granero-Molina J, Torrente-Sánchez MJ, Ferrer-Márquez M, Hernández-Padilla JM, Sánchez-Navarro M, Ruiz-Muelle A, Ruiz-Fernández MD, Fernández-Sola C (2021). Sexuality amongst heterosexual women with morbid obesity in a bariatric surgery programme: a qualitative study. J Adv Nurs.

[30] Granero-Molina J, Torrente-Sánchez MJ, Ferrer-Márquez M, Hernández-Padilla JM, Ruiz-Muelle A, López-Entrambasaguas OM, Fernández-Sola C (2020). Sexuality amongst heterosexual men with morbid obesity in a bariatric surgery programme: a qualitative study. J Clin Nurs.

[31] Lindberg S, Wennström B, Larsson AK (2022). Facing an unexpected reality - oscillating between health and suffering 4–6 years after bariatric surgery. Scand J Caring Sci.

[32] Merleau Ponty (2013). M. Phenomenology of perception.

[33] Tong A, Sainsbury P, Craig J (2007). Consolidated criteria for reporting qualitative research (COREQ): a 32-item checklist for interviews and focus groups. Int J Qual Health Care.

[34] Fernández-Sola C. Análisis de datos cualitativos [Qualitative data análisis] In Comprender para cuidar. Avances en Investigación Cualitativa en Ciencias de la Salud; Fernández-Sola, C., Granero-Molina, J. Hernández-Padilla, J.M, editors Servicio Publicaciones Universidad de Almería (Colección Ciencias de la Salud, Almería, 2019, pp. 239–261.

[35] Lincoln YS, Guba EG (1985). Naturalistic inquiry.

[36] Nilsson-Condori E, Järvholm S, Thurin-Kjellberg A, Hedenbro J, Friberg B (2020). A new beginning: young women’s experiences and sexual function 18 months after bariatric surgery. Sex Med.

[37] Oliveira CFA, Dos Santos PO, de Oliveira RA, Leite-Filho H, de Almeida AF, Bagano GO, Lima EB, Miranda EP, de Bessa J, Arroso U (2019). Changes in sexual function and positions in women with severe obesity af-ter bariatric surgery. Sex Med.

[38] Souza ALL, de Souza PM, Mota BEF, Xavier CLF, Santiago FG, Oliveira JS, Borges SA, Bearzoti E, Gama EF, Souza GGL (2023). Ghost fat: altered female body perception after bariatric surgery. Percept Mot Skills.

[39] Cherick F, Te V, Anty R, Turchi L, Benoit M, Schiavo L, Iannelli A (2019). Bariatric surgery significantly improves the quality of sexual life and self-esteem in morbidly obese women. Obes Surg.

[40] Abdelsamea GA, Amr M, Tolba AMN, Elboraie HO, Soliman A, Al-Amir B, Ali F, Osman DA. Impact of weight loss on sexual and psychological functions and quality of life in females with sexual dysfunction: a forgotten avenue. Front Psychol. 2023;1(14):1090256. 10.3389/fpsyg.2023.1090256 PMC9929060 36818091

[41] Pokorski M, Głuch A (2022). Perception of well-being and quality of life in obese patients after bariatric surgery. Adv Exp Med Biol.

[42] Wingfield LR, Kulendran M, Laws G, Chahal H, Scholtz S, Purkayastha S (2016). Change in sexual dysfunction following bariatric surgery. Obes Surg.

[43] Bramming M, Hviid SS, Becker U, Jørgensen MB, Neermark S, Bisgaard T, Tolstrup JS (2021). Changes in relation-ship status following bariatric surgery. Int J Obes (Lond).

[44] Treacy PJ, Mazoyer C, Falagario U, Iannelli A (2019). Sexual activity after bariatric surgery: a prospective monocentric study using the PISQ-IR Questionnaire. J Sex Med.

[45] Baillot A, Brunet J, Lemelin L, Gabriel SA, Langlois MF, Tchernof A, Biertho L, Rabasa-Lhoret R, Garneau PY, Aimé A, Bouchard S, Romain AJ, Bernard P (2023). Factors associated with excess skin after bariatric surgery: a mixed-method study. Obes Surg.

[46] Perdue TO, Schreier A, Neil J, Carels R, Swanson MA (2020). Concept analysis of disturbed body image in bariatric surgery patients. Int J Nurs Knowl.

[47] Alleva JM, Atkinson MJ, Vermeulen W, Monpellier VM, Martijn C (2023). Beyond body size: focusing on body func-tionality to improve body image among women who have undergone bariatric surgery. Behav Ther.

[48] Walsh Ó, Dettmer E, Regina A, Dentakos S, Christian J, Hamilton J, Toulany A (2022). Teenagers are into perfect-looking things’: dating, sexual attitudes and experiences of adolescents with severe obesity. Child Care Health Dev.

